# A Novel Electrochemical Sensor Based on r-GO@SiC Nanocomposite Materials for the Highly Sensitive Detection of Metronidazole

**DOI:** 10.3390/molecules31162846

**Published:** 2026-08-14

**Authors:** Yrysgul Bakytkarim, Zhazira Mukatayeva, Dinara Zhetpisbay, Nurgul Shadin, Ainur Yerezhepova, Yerzhan Imanbayev, Ainura Rakhimova, Yernar Kanzharkhan

**Affiliations:** 1 Department of Chemistry, Faculty of Natural Sciences and Geography, Abai Kazakh National Pedagogical University, 13, Dostyk Ave., Almaty 050010, Kazakhstan; rysgul_01_88@mail.ru (Y.B.); yerezhepova.ainur@gmail.com (A.Y.); 2 Department of Biochemistry, Asfendiyarov Kazakh National Medical University, 94, Tole Bi Street, Almaty 050000, Kazakhstan; zhetpisbay.d@kaznmu.kz; 3 Laboratory of Petrochemical Procrsses, Institute of Combustion Problems, 172, Bogenbai Batyr Str., Almaty 050012, Kazakhstan; erzhan.imanbayev@mail.ru (Y.I.); ainura_302015@mail.ru (A.R.); erxank@mail.ru (Y.K.)

**Keywords:** silicon carbide, reduced graphene oxide, SiC/rGO nanocomposite, electrochemical sensor, glassy carbon electrode, metronidazole, cyclic voltammetry, electrochemical impedance spectroscopy

## Abstract

In this study, a novel SiC/rGO nanocomposite-modified glassy carbon electrode (SiC/rGO/GCE) was developed as a simple, cost-effective, and efficient electrochemical platform for metronidazole (MTZ) detection. The combination of silicon carbide (SiC) and reduced graphene oxide (rGO) provides a favorable interface with a high electroactive surface area, efficient electron transfer, and enhanced electrocatalytic activity. The mor-phology and surface characteristics of the modified electrode were investigated by scan-ning electron microscopy (SEM), while its electrochemical properties were evaluated by cyclic voltammetry and electrochemical impedance spectroscopy. The results confirmed successful electrode modification and improved electron-transfer kinetics compared with the bare GCE. The main experimental parameters were systematically optimized, with the optimum conditions established at pH 10, an accumulation time of 300 s, and an accu-mulation potential of 0.5 V. Under these conditions, the SiC/rGO/GCE exhibited a broad linear response to MTZ over the concentration range of 5–5000 µmol/dm^3^, with a detection limit of 0.5 µmol/dm^3^ (S/N ≥ 3). The enhanced analytical performance is attributed to the synergistic contribution of rGO and SiC, where rGO promotes rapid electron transport and provides a large electroactive surface, while SiC contributes additional active sites and structural stability. The sensor was successfully applied to pharmaceutical samples, providing recoveries of 99.85–102.29% with RSD values below 2%. These results demon-strate the practical potential of the proposed sensor for reliable MTZ determination. Fur-ther validation in food and biological matrices and comparison with reference chromato-graphic methods will be necessary to establish its broader analytical applicability.

## 1. Introduction

The safety of food is a vital component of public health. In recent years, the presence of antibiotic residues in animal products—particularly meat, milk, eggs and honey—has become a pressing issue. Antibiotics are effective medicines for treating bacterial infections in both humans and animals; they are widely used to treat such infections and are regarded as one of the most significant achievements of modern medicine [1,2]. However, the misuse and overuse of antibiotics can lead to the accumulation of antibiotic residues in food, posing a serious threat to human health. Metronidazole (MTZ, 1-[2-hydroxyethyl]-2-methyl-5-nitroimidazole) is a widely used antibiotic. It is a synthetic antimicrobial agent belonging to the nitroimidazole class, exhibiting high antibacterial activity against anaerobic bacteria, trichomonads, amoebae and Giardia lamblia. Metronidazole is widely used to treat a variety of conditions, such as endocarditis, Crohn’s disease, gynaecological infections and respiratory tract infections [3,4]. However, excessive intake of this drug can have adverse effects on the human body. In particular, the risk of toxic reactions increases when the daily dose exceeds 2 g, and long-term use may be associated with carcinogenic effects [5]. Therefore, the development of a high-precision, high-sensitivity method for detecting metronidazole residues in food is one of the most pressing scientific tasks.

Various analytical techniques have been employed to determine metronidazole (MTZ) in a range of matrices, such as spectrophotometry, liquid chromatography–electrospray ionisation tandem mass spectrometry, chromatography, fluorimetry, chemiluminescence and ultra-high-performance liquid chromatography-tandem mass spectrometry [6,7,8,9,10,11,12,13,14]. Although these methods offer high sensitivity and selectivity, they also suffer from drawbacks such as complex operational procedures, time-consuming sample preparation, lengthy analysis times, high equipment costs, and the need for skilled operators [15]. In contrast, voltammetry offers advantages such as cost-effectiveness, speed, ease of operation, high specificity, high sensitivity, portability, and potential for miniaturisation [16,17,18]. Consequently, this study selected electrochemical methods to determine metronidazole residues in food samples. Among these, square-wave voltammetry (SWV) was chosen due to its numerous advantages, including ultra-rapid analysis, minimal sample requirements, strong background discrimination capability, and high specificity [19,20,21,22].

Carbonaceous electrodes are widely employed in the fabrication of electrochemical sensors owing to their broad potential window, low background current, excellent electrical conductivity, cost-effectiveness, and ease of surface modification [23,24]. Among carbon-based nanomaterials, graphene has attracted significant attention due to its extraordinary physicochemical properties, including high mechanical strength, large specific surface area, superior electron mobility, excellent thermal conductivity, biocompatibility, and chemical stability. Reduced graphene oxide (r-GO), a derivative of graphene, is particularly attractive for electrode modification because it retains many of graphene’s desirable properties while offering abundant defect sites and residual oxygen-containing functional groups that facilitate surface functionalization and enhance electrocatalytic activity [25]. The incorporation of r-GO into electrochemical sensing platforms significantly improves electron transfer kinetics, increases the electroactive surface area, minimizes electrode fouling, and enhances analytical sensitivity. Furthermore, its porous structure promotes efficient diffusion of analyte molecules toward the electrode surface, resulting in improved detection performance. Consequently, r-GO-based electrochemical sensors have demonstrated remarkable analytical characteristics, including low detection limits, wide linear ranges, and high selectivity toward various target compounds.

Recent studies have revealed the potential of rGO in the fields of catalysis, electromagnetic shielding, and sensor technology. Porous, nitrogen-doped rGO has demonstrated high catalytic activity for the reduction of aromatic nitrophenols, which is attributed to the synergistic interaction between nitrogen-containing functional groups and the porous structure of graphene [26]. Furthermore, the incorporation of rGO into α-FeOOH enabled the development of PVA/rGO/α-FeOOH composites with enhanced electrical and thermal conductivity, as well as improved electromagnetic interference shielding properties [27]. Similarly, PEDOT/N-rGO/Fe_3_O_4_ composites containing nitrogen-doped rGO and Fe_3_O_4_ demonstrated high electrical conductivity and excellent electromagnetic shielding performance [28]. In addition to these applications, rGO has also attracted significant interest as an effective sensor material due to its large specific surface area, abundance of surface functional groups, and favorable charge transport properties. Its integration with semiconductor nanoparticles, such as CeO_2_, can enhance analyte adsorption and facilitate interphase charge transfer, thereby improving NO_2_ detection performance [29]. Furthermore, rGO doped with heteroatoms exhibits an enhanced response to trace concentrations of NO_2_ due to the formation of additional active sites and functional linkers [30].

Building on these advantages, combining carbon nanomaterials with semiconductor nanostructures can generate synergistic effects that further enhance sensor performance. Silicon carbide (SiC) is considered one of the most promising semiconductor materials for electrochemical applications due to its exceptional physicochemical properties. SiC exhibits high thermal stability, excellent chemical inertness, superior mechanical strength, wide band gap, low toxicity, corrosion resistance, and long-term operational stability under harsh environmental conditions. In addition, SiC possesses favorable electron transport characteristics and high surface durability, making it an attractive candidate for advanced electrochemical sensing platforms [31,32]. The integration of SiC nanocomposites with r-GO provides several advantages. The conductive r-GO network facilitates rapid electron transport, while SiC nanoparticles contribute additional active sites and structural stability. This combination prevents the agglomeration of graphene sheets, increases the accessible surface area, accelerates charge-transfer processes, and improves electrocatalytic activity. Moreover, the synergistic interaction between r-GO and SiC enhances sensitivity, repeatability, and long-term stability of the sensor. Therefore, the development of r-GO/SiC nanocomposite-modified electrodes represents an effective strategy for constructing highly sensitive and reliable electrochemical sensors.

In this work, a novel r-GO/SiC nanocomposite-modified electrode was successfully developed for the electrochemical determination of metronidazole. The conductive r-GO framework facilitates rapid electron transfer, whereas SiC nanoparticles provide structural robustness, additional catalytic sites, and long-term stability. The synergistic effect between these materials significantly enhances the electrochemical performance of the sensor, resulting in improved sensitivity, selectivity, and analytical reliability. The proposed sensor offers a simple, cost-effective, and efficient platform for monitoring metronidazole residues in food matrices.

## 2. Results and Discussion

### 2.1. Structural and Morphological Characterization of the SiC/rGO Nanocomposite

**Figure 1:**
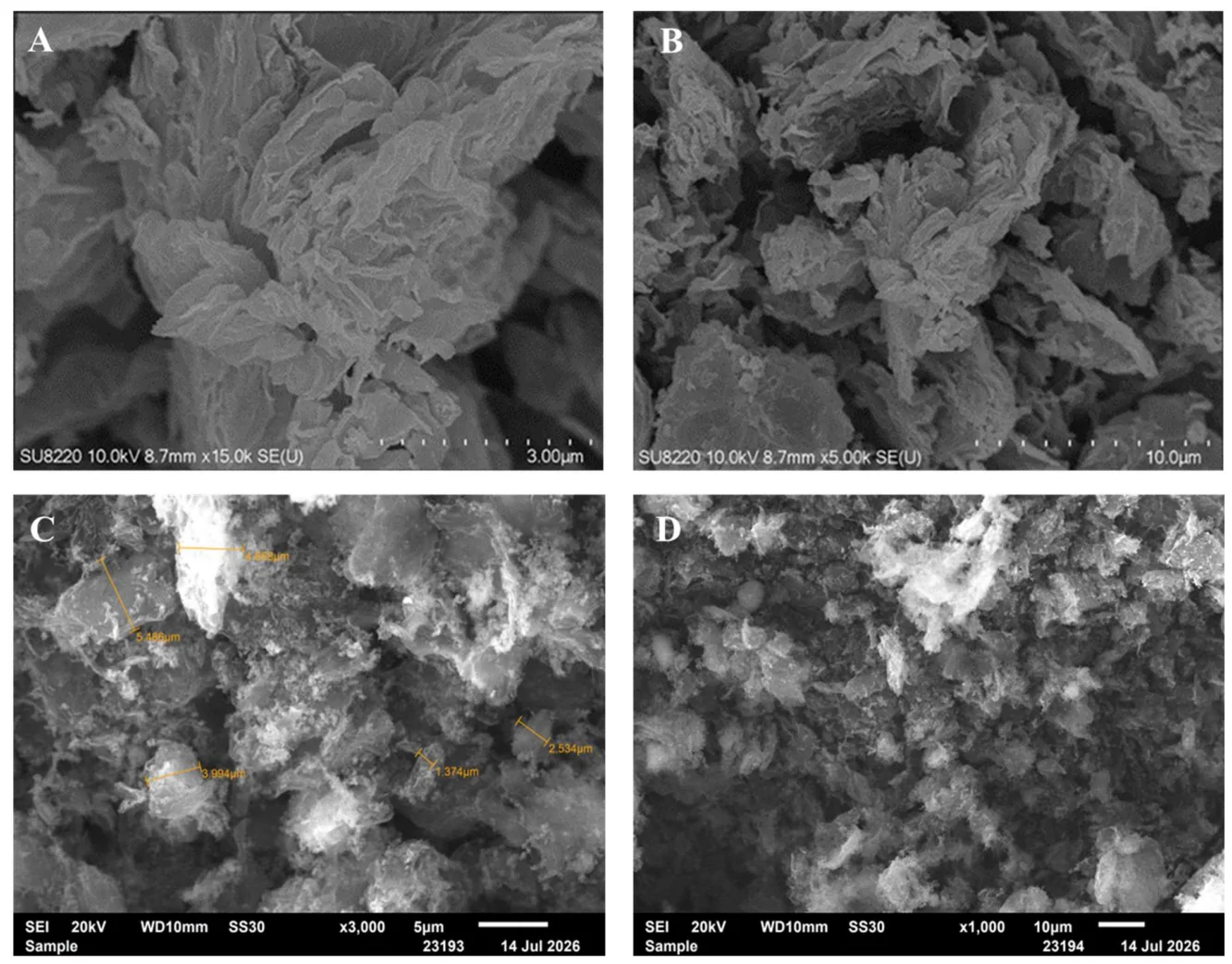

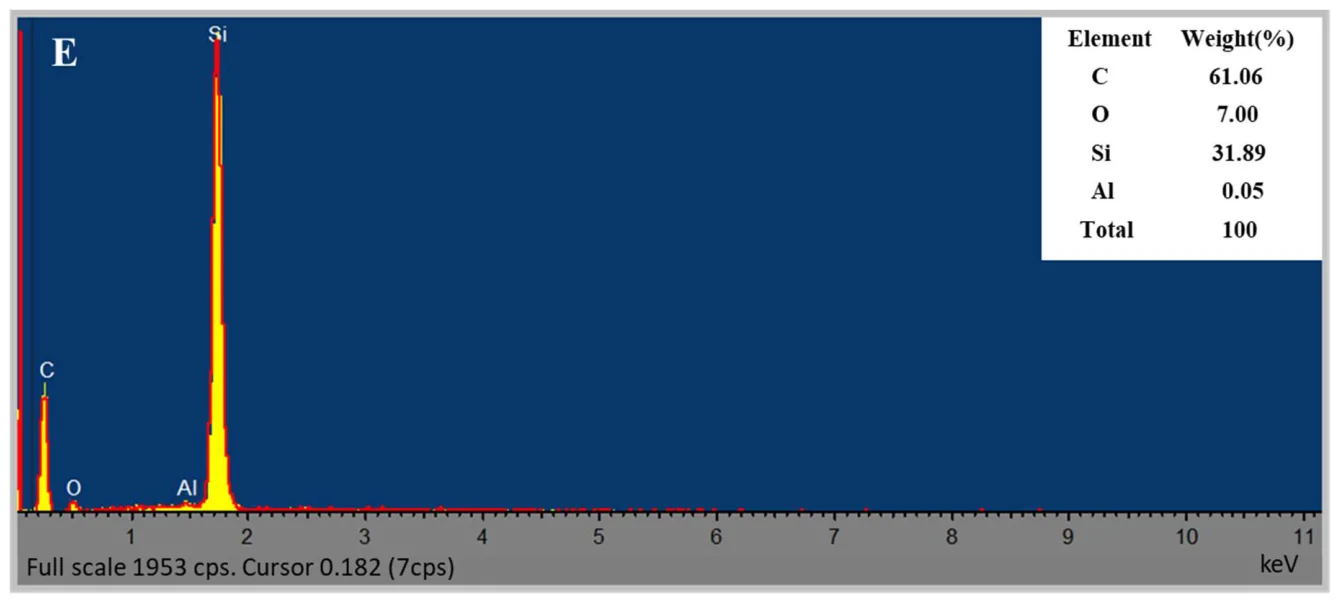
Morphological characterization and elemental composition of the synthesized SiC/rGO nanocomposite: (**A**–**D**) SEM images at different magnifications; (**E**) EDS spectrum.

Scanning electron microscopy (SEM) and energy-dispersive X-ray spectroscopy (EDS) were employed to investigate the morphological structure and elemental composition of the synthesized SiC/rGO nanocomposite. As shown in Figure 1A–D, the SEM images reveal that SiC particles are successfully dispersed within the porous rGO matrix, forming a heterogeneous architecture. At low magnification (10.0 µm scale), the graphene sheets appear overlapped and folded, resulting in a characteristic crumpled and multilayered morphology with abundant pores. Higher-magnification SEM images (3.00 µm scale) further demonstrate that the rGO nanosheets possess a thin, wrinkled, and multilayered structure, while the SiC particles are relatively uniformly distributed throughout the graphene matrix, with only slight aggregation. The SEM analysis revealed a broad SiC particle/agglomerate size distribution ranging from 1.07 to 3.74 µm, with an average size of 2.12 ± 1.06 µm. The observed size variation can be mainly attributed to the aggregation of SiC nanoparticles within the rGO matrix. The close interfacial contact between SiC and rGO suggests favorable interactions between the two components, which may facilitate electron transport and enhance the electrochemical activity of the nanocomposite. These structural characteristics provide a large accessible surface area and numerous electrochemically active sites, thereby contributing to the enhanced sensing performance of the SiC/rGO nanocomposite. Overall, the SEM observations confirm the successful synthesis of the SiC/rGO nanocomposite and demonstrate its promising potential as an electrode material for electrochemical sensing applications.

Figure 1E presents the EDS spectrum of the synthesized SiC/rGO nanocomposite. The elemental analysis confirms the presence of carbon (61.06 wt%), silicon (31.89 wt%), oxygen (7.00 wt%), and a trace amount of aluminum (0.05 wt%). The high carbon content originates from the reduced graphene oxide (rGO), while the strong silicon signal confirms the successful incorporation of SiC into the composite. The low oxygen content indicates the effective reduction of graphene oxide, and the trace aluminum is attributed to residual impurities or the sample preparation process. These results verify the successful synthesis of the SiC/rGO nanocomposite.

### 2.2. Comparative Analysis of Electrochemical Properties of Modified Electrodes

**Figure 2:**
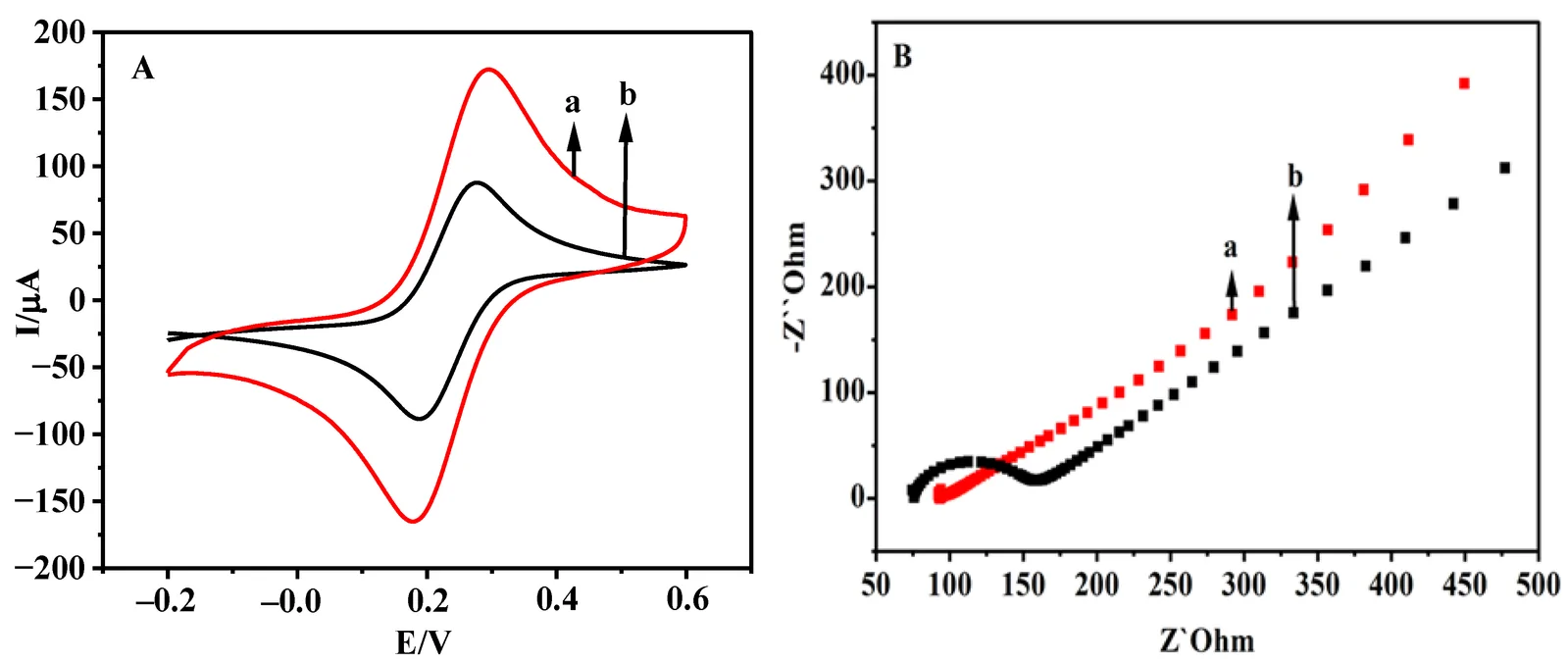
Cyclic voltammetry (**A**) and electrochemical impedance spectroscopy (EIS) (**B**) of bare GCE (curve b), SiC/rGO/GCE (curve a) obtained in the presence of 5 µmol/dm^3^ K_3_[Fe(CN)_6_]/K_4_[Fe(CN)_6_] in 0.1 M KCl solution.

Cyclic voltammograms of bare glassy carbon electrode (GCE) and GCE electrodes modified with SiC/rGO-based nanocomposite material in the presence of 5 µmol/dm^3^ K_3_Fe(CN)_6_/K_4_[Fe(CN)_6_] system in 0.1 M KCl solution were studied. In Figure 2A, in the case of bare GCE (curve b), oxidation and reduction peaks were observed at potentials of 0.29 V and 0.17 V, respectively. However, after the electrode surface was treated with SiC/rGO nanomaterial (curve a), a clear increase in current values corresponding to redox processes was detected. This phenomenon is explained by the high electrical conductivity of the SiC/rGO nanocomposite and an increase in the active surface area of the electrode, as a result of which the electron exchange process occurs more efficiently. Furthermore, the electrochemical surface area (ECSA) was calculated using the Randles–Ševčík equation: Ip = 2.69 × 10^5^ n^3/2^AD^1/2^Cν^1/2^. Using ferricyanide as the redox probe, the calculated ECSA of the SiC/rGO/GCE was 0.1828 cm^2^, which is higher than that of the bare GCE (0.0707 cm^2^), confirming the increased electrochemically active surface area after modification.

Electrochemical impedance spectroscopy (EIS) is one of the most effective methods for evaluating changes occurring at the electrode surface. Figure 2B presents the EIS results obtained for the bare GCE and the SiC/rGO-modified GCE in a 5 μmol dm^−3^ K_3_[Fe(CN)_6_]/K_4_[Fe(CN)_6_] solution containing 0.1 M KCl. The bare GCE (curve b) exhibited a relatively high charge-transfer resistance, as indicated by the fitted Rct value of 75 Ω. However, after modification of the electrode surface with the SiC/rGO nanocomposite (curve a), the Nyquist plot showed a smaller semicircular region and a more pronounced linear portion, indicating a decrease in the charge-transfer resistance of the system. The SiC/rGO nanocomposite-modified electrode provided enhanced electrical conductivity and facilitated electron transfer at the electrode/electrolyte interface. Therefore, the CV and EIS results collectively demonstrate the successful fabrication of the SiC/rGO-based sensor and confirm the improvement of its electrochemical properties.

### 2.3. Electrochemical Behavior of Metronidazole on Different Modified Electrodes

**Figure 3:**
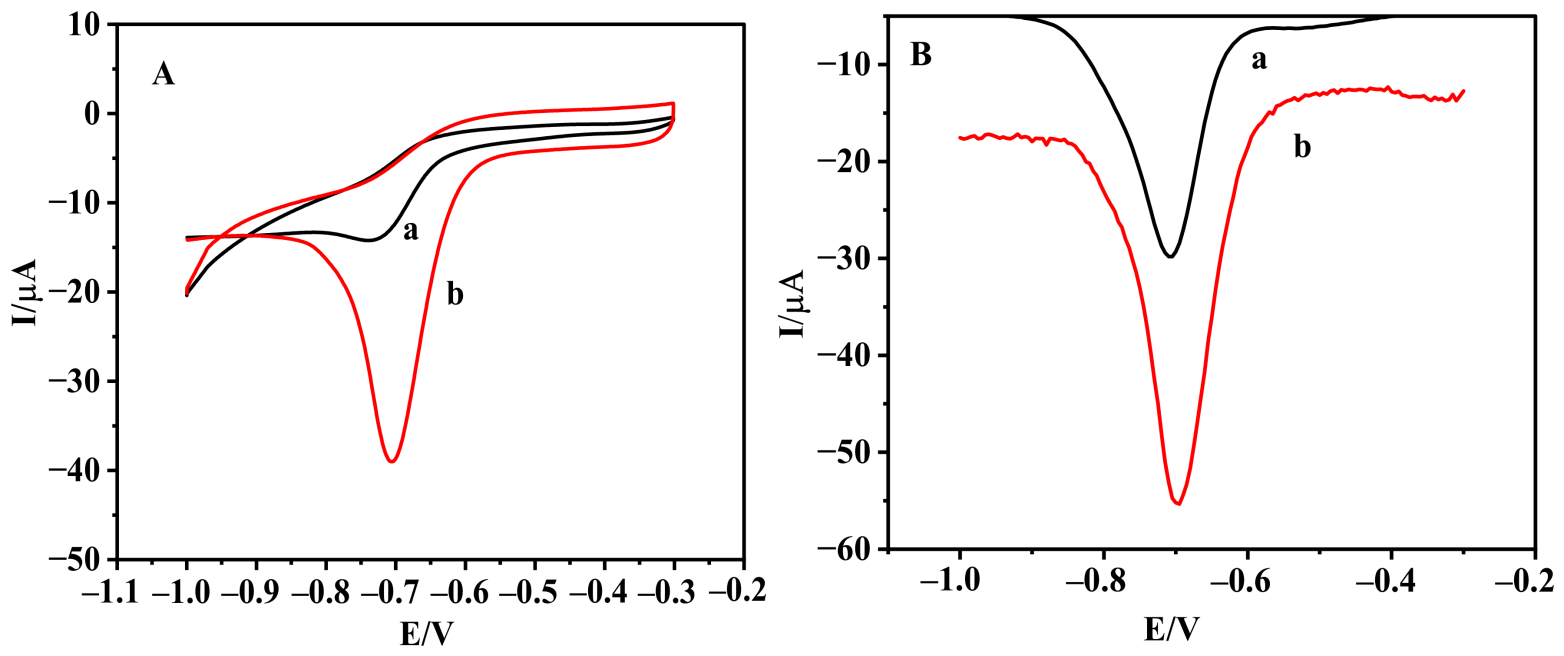
(**A**) Cyclic voltammetry and (**B**) differential pulse voltammetry of 500 µM/dm^3^ metronidazole in 0.1 M PBS (pH = 10.0) buffer solution on (a) bare GCE and (b) SiC/rGO nanocomposites/GCE surface.

The electrochemical behavior of metronidazole was investigated using a glassy carbon electrode (GCE) modified with the SiC/rGO nanocomposite. Figure 3A shows the cyclic voltammograms recorded for bare GCE and SiC/rGO/GCE in a 0.1 M PBS buffer solution (pH 10.0) containing 500 μmol dm^−3^ metronidazole. For the bare GCE (curve a), the reduction peak of metronidazole was observed at a peak current (Ip) of approximately 11.46 μA and a peak potential (Ep) of approximately −0.738 V. In contrast, for the SiC/rGO-modified electrode (curve b), the reduction peak current increased significantly to approximately 35.26 μA, while the peak potential shifted positively to approximately −0.707 V.

These changes can be attributed to the high electrical conductivity of the SiC/rGO nanocomposite and the increased electroactive surface area of the electrode, which promote the electron-transfer process and facilitate the electrochemical reduction of metronidazole. The differential pulse voltammetry (DPV) results shown in Figure 3B are consistent with the cyclic voltammetry data. The enhanced peak current obtained with the SiC/rGO/GCE further demonstrates the electrocatalytic activity of the nanocomposite toward metronidazole reduction. These results indicate that the SiC/rGO-modified GCE exhibits high sensitivity and excellent electrochemical performance for the determination of metronidazole.

### 2.4. Effect of Scanning Speed

**Figure 4:**
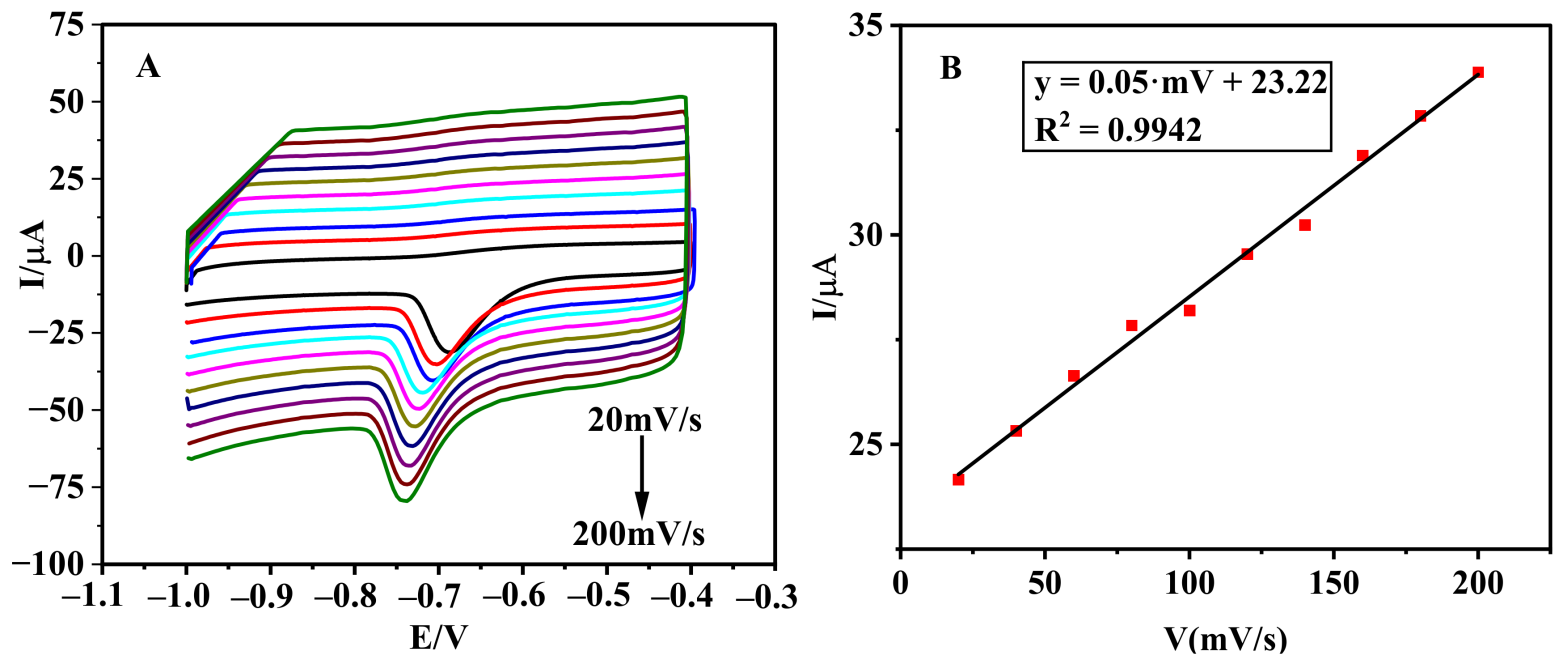
(**A**) Cyclic voltammetry plots of 500 µM/dm^3^ metronidazole on SiC/rGO/GCE surface in 0.1 M PBS (pH = 10.0) buffer solution at different scan rates. (**B**) Relationship between reduction peak currents and scan rates.

**Figure 5:**
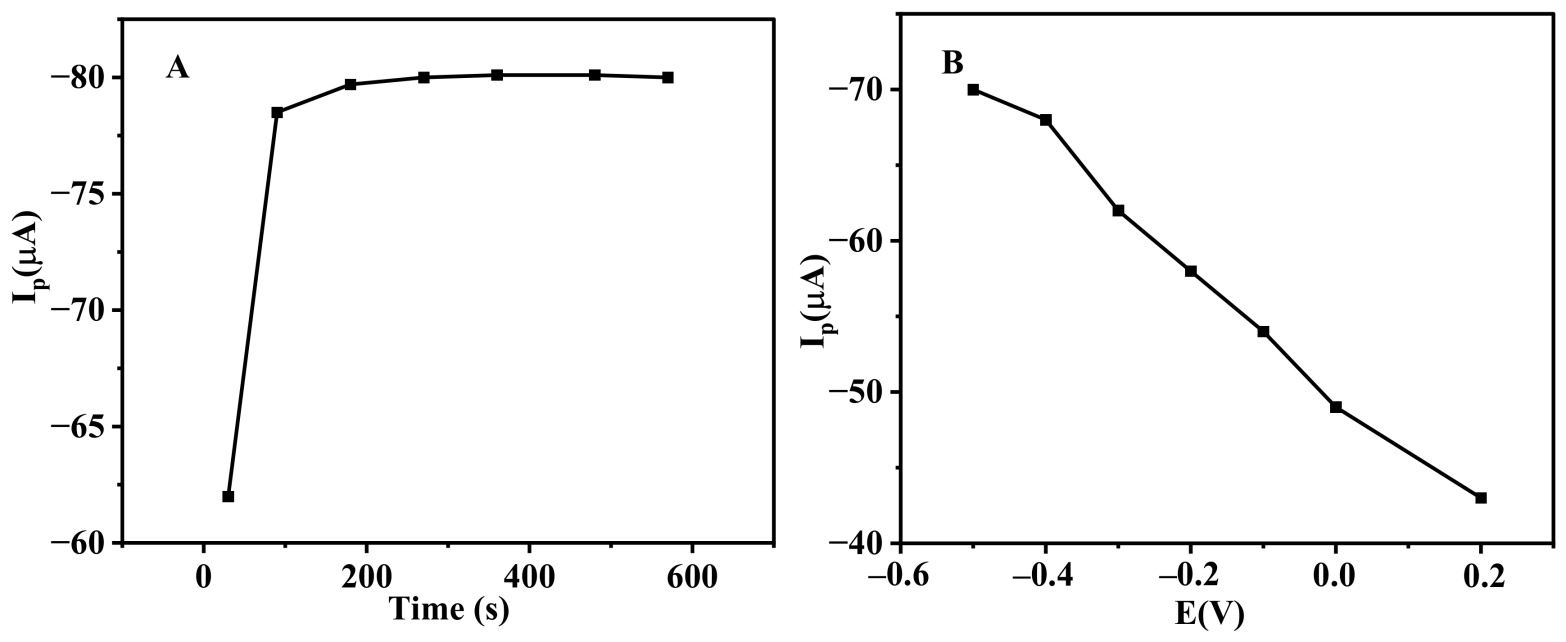
Effects of accumulation time (**A**) and accumulation potential (**B**) on the reduction peak current of metronidazole in 0.1 M PBS (pH = 10.0) solution in the SiC/rGO/GCE-based electrochemical sensor.

The effect of metronidazole on the electrochemical behavior was studied at different scanning speeds. Figure 4A shows the cyclic voltammograms for the SiC/rGO/GCE electrode in 500 µmol/dm^3^ metronidazole solution, with the reduction current continuously increasing with increasing scanning speed. The results showed a linear relationship between the reduction peak currents and the scanning speed. Figure 5B shows the linear regression equation of the form Ip (µA) ≈ 0.05·ν (mV/s) + 23.22 (R^2^ = 0.9942), which indicates a linear relationship with high accuracy. The results of the study indicate that the electrochemical process of metronidazole occurs through adsorption, that is, the substance accumulates on the electrode surface and reacts.

### 2.5. Effect of Accumulation Time and Accumulation Potential

The effects of accumulation time and accumulation potential on the determination of metronidazole were investigated using differential pulse voltammetry (DPV).

As shown in Figure 5A, the reduction peak current at an accumulation potential of 0.5 V gradually increased with the increase in accumulation time and reached a maximum value at 300 s. However, the reduction peak current remained unchanged after 300 s due to the saturation of the surface active catalytic sites of the SiC/rGO nanocomposites. Therefore, 300 s was used as the optimal accumulation time. The effect of accumulation potential on the reduction peak current of metronidazole was further investigated with the above-determined optimal accumulation time. As shown in Figure 5B, the reduction peak current gradually decreased with the increase in accumulation potential; therefore, 0.5 V was selected as the accumulation potential.

### 2.6. Effect of pH

**Figure 6:**
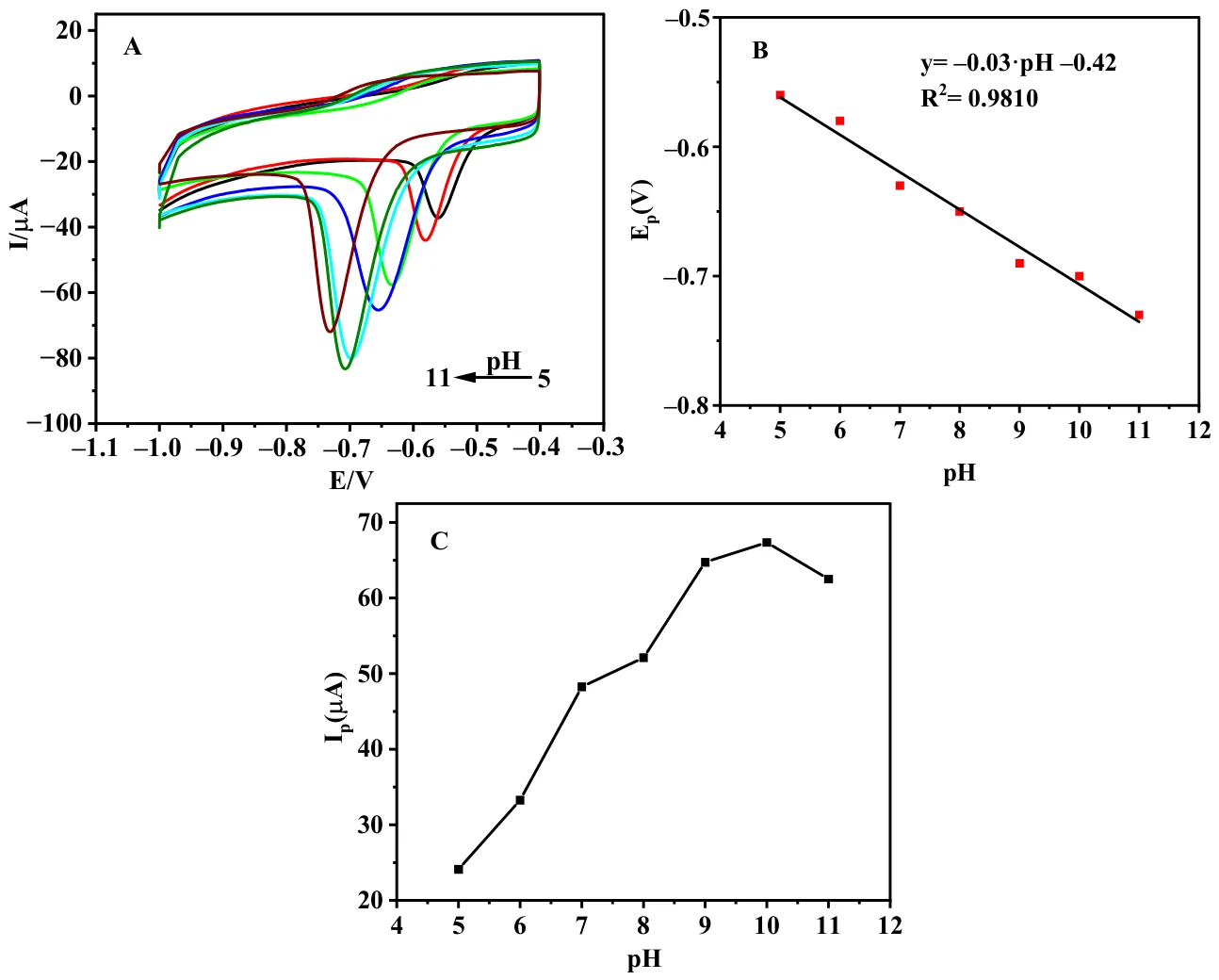
(**A**) Cyclic voltammetry plots of 500 µM/dm^3^ metronidazole in 0.1 M PBS (pH = 10.0) buffer solution at different pH values on the SiC/rGO nanocomposites/GCE surface. (**B**) Dependence of reduction peak potentials on pH values and (**C**) dependence of reduction peak currents on pH values.

The effect of 500 µmol/dm^3^ metronidazole in 0.1 M PBS buffer solution in the pH range of 5.0 to 11.0 on the electrochemical response of SiC/rGO/GCE was studied by cyclic voltammetry. Figure 6A shows the cyclic voltammograms at different pH values, and a single reduction peak was observed in the figure at all pH values, with a reduction potential. The reaction mechanism is shown below:

$$R-{NO}_{2}+e^{-}\to R-{NO}_{2}\cdot^{-}$$

$$R-{NO}_{2}\cdot^{-}+\;4H^{+}+3e^{-}\to R-NHOH+H_{2}O$$

This can be explained by the fact that the reduction of the nitro group of metronidazole occurs as a single-step reduction process with a total addition of 4***e*^−^**. That is, although the intermediate nitro radical (***R*** **−** ***NO*_2_*∙*^−^**) is formed, it is unstable and quickly reduced to hydroxylamine (***R − NHOH***) on the electrode surface. Figure 6B shows a linear relationship between the reduction peak potentials and the pH of the buffer solution in the range of 5.0–11.0, and the linear regression equation: Ep = −0.02893 pH—0.41714 (R^2^ = 0.9810), indicating different reaction mechanisms. In addition, as shown in Figure 6C, since the reduction peak current reaches its maximum value at pH 10.0, 10.0 was chosen as the most suitable pH value for the determination of metronidazole.

### 2.7. Quantitative Determination of Metronidazole

**Figure 7:**
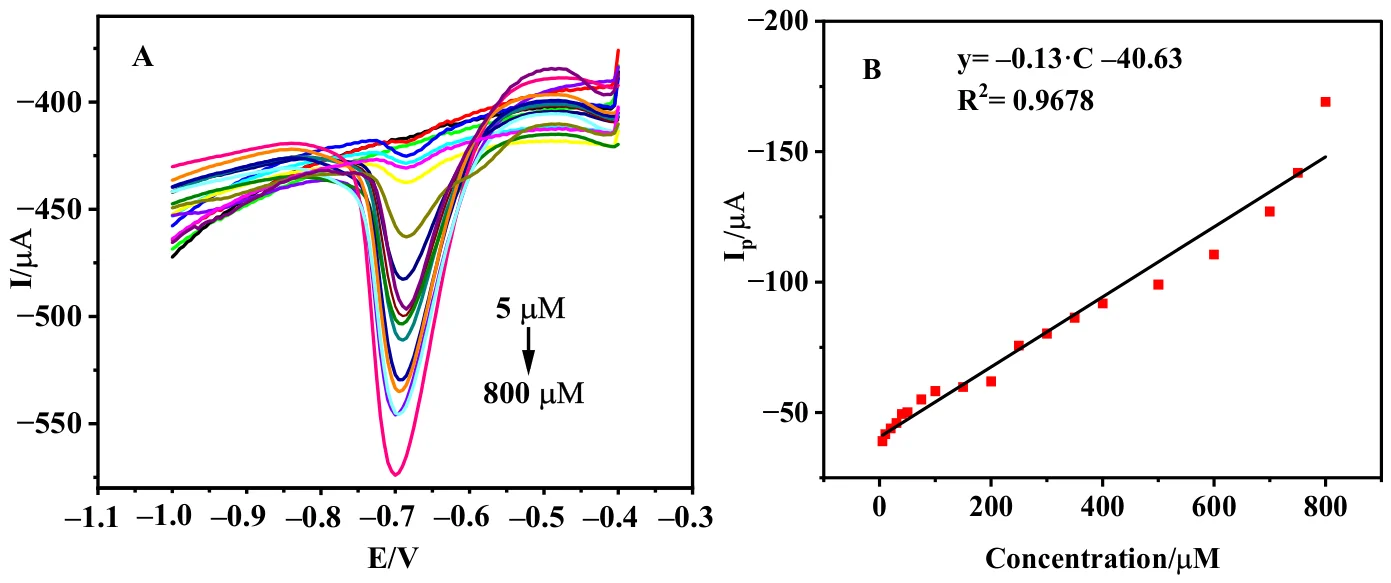
Differential pulse voltammetry of metronidazole in 0.1 M PBS (pH = 10.0) buffer solution at (**A**) concentrations of 5–800 µmol/dm^3^ on the SiC/rGO nanocomposites/GCE surface. (**B**) Linear relationships between reduction peak currents and concentrations in the range of 5–800 µmol/dm^3^.

**Figure 8:**
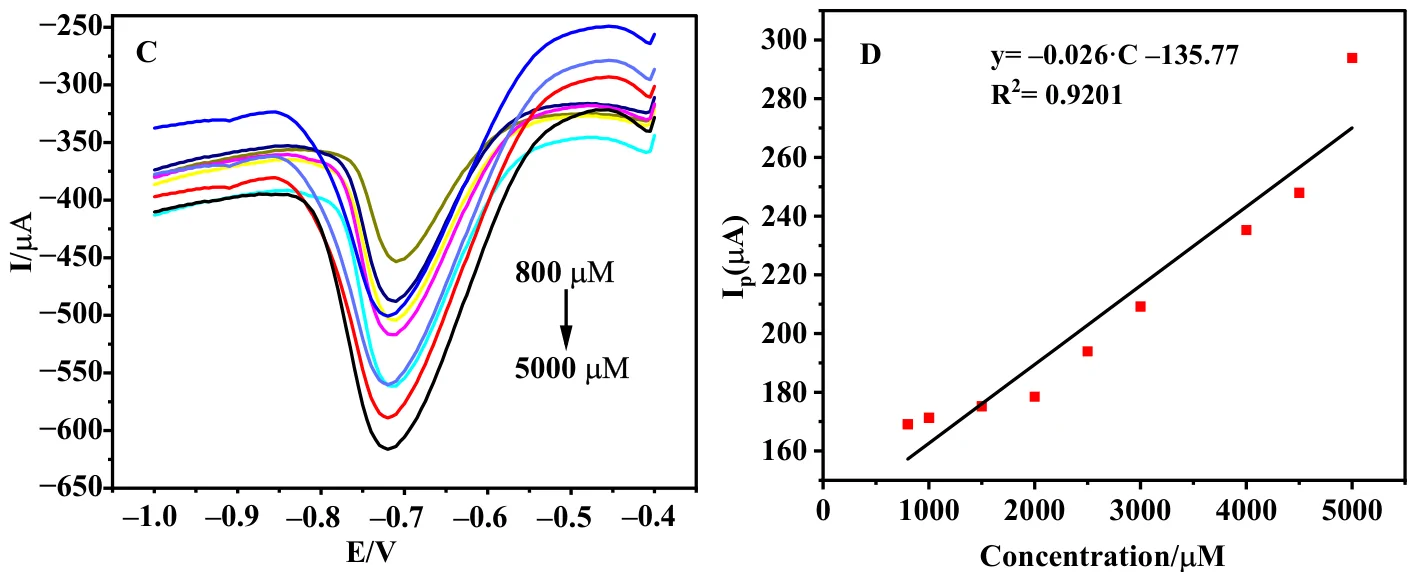
Differential pulse voltammetry of metronidazole in 0.1 M PBS (pH = 10.0) buffer solution (**C**) at concentrations of 800–5000 µmol/dm^3^ on SiC/rGO nanocomposites/GCE. (**D**) Linear relationships between reduction peak currents and concentrations in the range of 800–5000 µmol/dm^3^.

The determination of different concentrations of metronidazole was carried out by differential pulse voltammetry. As shown in Figure 7A and Figure 8C, the reduction peak currents of metronidazole on the SiC/rGO/GCE surface increased linearly in the concentration range of 5–800 µmol/dm^3^ and varied in the range of 800–5000 µmol/dm^3^, and their corresponding linear regression equations are shown in Table 1. The detection limit of metronidazole is 0.5 µmol/dm^3^ (S/N ≥ 3).

Also, compared with the most common electrochemical sensors for metronidazole detection recently, our proposed nanocomposite sensor can perform sensitive detection of metronidazole in a much wider linear range and with much lower detection limits (Table 2).

### 2.8. Stability and Selectivity of SiC/rGO/GCE

The selectivity, stability, and repeatability of the developed SiC/rGO/GCE sensor were evaluated to assess its suitability for metronidazole (MTZ) determination. The selectivity studies were performed in 0.1 M PBS (pH 10.0) containing 500 µmol/dm^3^ MTZ. The addition of various inorganic ions (K^+^, Mg^2+^, Zn^2+^, Na^+^, Ca^2+^, PO_4_^3−^, SO_4_^2−^, F^−^, CO_3_^2−^, NO_3_^−^, and Cl^−^) at 100-fold higher concentrations caused a signal deviation of less than 3.5%. Similarly, common organic compounds, including oxalic acid, ascorbic acid, glucose, citric acid, cysteine, alanine, and tartaric acid, at 10-fold higher concentrations resulted in signal deviations of less than 6.5%. These results demonstrate the good selectivity of the SiC/rGO/GCE toward MTZ in the presence of potential interfering substances.

The storage stability of the modified electrode was also evaluated. After storage at 4 °C for one month, the reduction peak current retained 96.2% of its initial value, confirming the good stability of the sensor. The repeatability was evaluated using four independently prepared modified electrodes. The relative standard deviation (RSD) of the measured responses for metronidazole was 2.5%, indicating high repeatability. Overall, the SiC/rGO/GCE sensor shows great potential for metronidazole detection due to its high selectivity, good stability, and excellent repeatability.

### 2.9. Detection of Real Samples

The practical analytical application of the SiC/rGO nanocomposites/GCE sensor was evaluated by the detection of metronidazole in real pharmaceutical samples using the standard addition method. All measurements were performed in triplicate. As shown in Table 3, the recoveries in real samples ranged from 99.85% to 102.29%, and the relative standard deviation (RSD) values were less than 2%, indicating that the proposed sensor can be effectively used for the accurate determination of metronidazole in real samples.

## 3. Materials and Methods

### 3.1. Chemicals and Reagents

Metronidazole (99%, analytical grade) was purchased from Macklin Biochemical Co., Ltd. (Shanghai, China). Drug samples were obtained from Huayueyang Biotechnology Co., Ltd. (Beijing, China). All other reagents were of analytical grade and used without further purification. A 0.1 M phosphate-buffered solution (PBS) was prepared by mixing sodium dihydrogen phosphate (NaH_2_PO_4_), disodium hydrogen phosphate (Na_2_HPO_4_), and potassium chloride (KCl), and the pH was adjusted to 10.0 using hydrochloric acid (HCl) or sodium hydroxide (NaOH) solution. All aqueous solutions were prepared using double-distilled water.

### 3.2. Synthesis of Silicon Carbide and Reduced Graphene Oxide Composite

In the preparation of the SiC/rGO composite material, SiC and rGO were used in an equal mass ratio of 1:1. First, 0.0045 g of SiC and 0.0045 g of rGO were accurately weighed using an analytical balance and mixed thoroughly to obtain the SiC/rGO composite powder. Subsequently, 1 mL of ethanol and 2 mL of distilled water were added to the obtained SiC/rGO powder. The resulting mixture was stirred and sonicated in an ultrasonic bath for 30 min at 25 °C to ensure homogeneous dispersion of the SiC and rGO components and to obtain a stable SiC/rGO suspension.

To ensure complete dispersion of the particles, the suspension was treated in an ultrasonic bath. This process reduced the agglomeration of the materials and promoted the formation of a stable composite structure. Subsequently, the suspension was further mixed using a magnetic stirrer to achieve a uniform dispersion.

The prepared SiC/rGO composite was used to modify the surface of the working electrode in subsequent electrochemical studies. The presence of rGO in the composite enhanced the electrical conductivity of the material, while SiC provided mechanical strength and structural stability. The combination of these properties contributed to the improved analytical performance of the electrochemical sensor.

### 3.3. Preparation of the SiC/rGO Nanocomposite-Modified GCE Sensor

During the preparation of the SiC/rGO nanocomposite-based sensor, the glassy carbon electrode (GCE) was first pretreated. To smooth the electrode surface and remove impurities, mechanical polishing was carried out using 0.3 μm and 0.05 μm alumina powders. After polishing, the electrode was ultrasonically cleaned in absolute ethanol and distilled water to completely remove any residual particles remaining on the surface. During the ultrasonic treatment, fine impurities and residues accumulated on the electrode surface were thoroughly eliminated, resulting in a uniform and clean surface. Subsequently, the electrode was dried under a nitrogen stream. As a result of these pretreatment procedures, the GCE was fully prepared for subsequent experimental applications.

**Figure 9:**
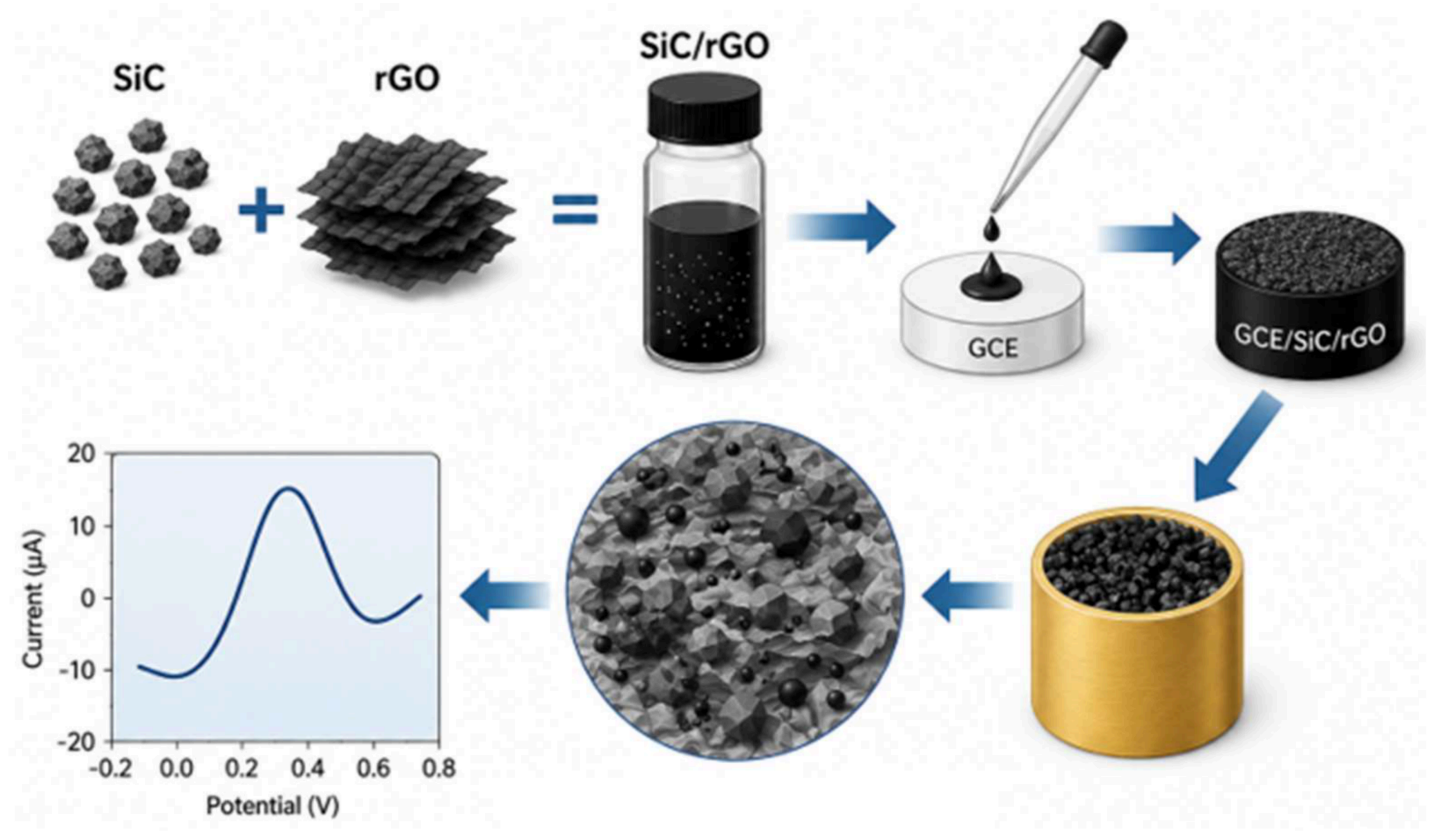
Fabrication process of SiC/rGO − based sensor.

As shown in Figure 9, during the modification phase, a homogeneous SiC/rGO suspension with a concentration of 0.5 mg/mL was prepared, and a volume of 5 µL was taken. The resulting suspension was dropwise applied to the electrode surface using a micropipette. The electrode was kept motionless for some time to ensure uniform distribution of the suspension on the electrode surface. Then the electrode was dried under an infrared lamp. During drying, the solvent evaporated, and the SiC/rGO nanocomposite formed a stable film on the electrode surface. As a result, a SiC/rGO/GCE sensor modified with the SiC/rGO nanocomposite was obtained.

### 3.4. Instruments and Methods

Cyclic voltammetry (CV), electrochemical impedance spectroscopy (EIS), and differential pulse voltammetry (DPV) measurements were carried out using an electrochemical workstation (CS350 Potentiostat/Galvanostat, Shanghai Chenhua Instrument Co., Ltd., Shanghai, China). A conventional three-electrode system was employed, consisting of a glassy carbon electrode (GCE, 3 mm in diameter) as the working electrode, a saturated calomel electrode (SCE) as the reference electrode, and a platinum wire as the counter electrode.

The electrochemical measurements were performed in a solution containing K_3_[Fe(CN)_6_]/K_4_[Fe(CN)_6_] and 0.1 M KCl. EIS measurements were conducted at the open-circuit potential over a frequency range from 0.1 Hz to 100 kHz with an AC amplitude of 5 mV.

The surface morphology of the prepared materials and modified electrodes was characterized using a field-emission scanning electron microscope (FE-SEM, Zeiss Ultra 55, Oberkochen, Germany).

## 4. Conclusions

In conclusion, a SiC/rGO nanocomposite was successfully synthesized and applied to fabricate a SiC/rGO-modified glassy carbon electrode (SiC/rGO/GCE) using a simple drop-casting method. SEM and electrochemical characterization confirmed the successful formation of the nanocomposite and its favorable electrochemical properties. Under the optimized conditions of pH 10, an accumulation time of 300 s, and an accumulation potential of 0.5 V, the proposed sensor exhibited a wide linear range of 5–5000 µmol/dm^3^ and a detection limit of 0.5 µmol/dm^3^ (S/N ≥ 3). The enhanced response is attributed to the synergistic effect of SiC and rGO, which improves electron transfer and increases the electroactive surface area. The sensor was successfully applied to pharmaceutical samples, providing recoveries of 99.85–102.29% with RSD values below 2%. Overall, the SiC/rGO/GCE provides a simple, reliable, and cost-effective approach for MTZ determination. Further validation in food and biological matrices, together with comparison with reference chromatographic methods, will be important for extending its practical analytical applicability.

## Figures and Tables

**Table 1:** Linear regression equations of metronidazole in different concentration ranges.

Equation	Linear Range (μM)	Regression Equation	R^2^
(1)	5–800	I_p_ (µA) = −0.13424 c (µM) − 40.6367	0.9678
(2)	800–5000	I_p_ (µA) = −0.02686 c (µM) − 135.7710	0.9201

**Table 2:** Comparative performance of SiC/rGO nanocomposites/GCE with other modified electrodes.

Electrode	LOD/(µmol/dm^3^)	Determination Range/(µmol/dm^3^)	References
GCE	1.100	2–600	[33]
DNA/GCE	1.000	1.0–54.3	[34]
P-AgSA-CE	0.600	2–100	[35]
Carbon fiber microdisk electrode	0.500	1–22	[36]
Cu-poly (Cys)/GCE	0.370	0.5–400	[37]
Graphene-ionic liquid/GCE	0.047	0.1–25	[38]
Carbon nanotubes/GCE	0.063	0.1–200	[39]
SiC/rGO/GCE	0.500	5–5000	This work

**Table 3:** Practical determination of metronidazole in real drug samples (Sample results are expressed as 95% confidence intervals).

Sample	Added Concentration (µmol/dm^3^)	Concentration Found in the Study (µmol/dm^3^)	Recovery Rate (Recovery, %)	Relative Standard Deviation (RSD, %)
1	500	511.447	102.29	1.25
2	5000	4992.772	99.85	1.14

## Data Availability

The original contributions presented in this study are included in the article. Further inquiries can be directed to the corresponding author.
